# Kidney transplantation in Icelandic patients, 2000–2019: are outcomes affected by low volume?

**DOI:** 10.3389/frtra.2024.1398444

**Published:** 2024-05-28

**Authors:** Thordur P. Palsson, Margret B. Andresdottir, Eirikur Jonsson, Johann Jonsson, Rafn Hilmarsson, Olafur S. Indridason, Runolfur Palsson

**Affiliations:** ^1^ Divison of Urology, Surgical Services, Landspitali University Hospital, Reykjavik, Iceland; ^2^ Division of Nephrology, Internal Medicine Services, Landspitali University Hospital, Reykjavik, Iceland; ^3^ Inova Transplant Center, Inova Fairfax Hospital, Fairfax, VA, United States; ^4^ Faculty of Medicine, School of Health Sciences, University of Iceland, Reykjavik, Iceland

**Keywords:** kidney graft, graft survival, graft function, deceased donor, living donor, low-volume center

## Abstract

**Background:**

In Iceland, a small number of kidney transplants from living donors (LDs) are performed at Landspitali University Hospital (LUH) in Reykjavik, while deceased donor transplants have until recently invariably been carried out abroad. In this study, we evaluated the outcome of kidney transplantation in Icelandic patients.

**Methods:**

This was a retrospective study that included all Icelandic residents who underwent kidney transplantation between 1 January 2000 and 31 December 2019. Data were obtained from the Icelandic End-Stage Kidney Disease Registry, medical records at LUH, and the Scandiatransplant database. The Chronic Kidney Disease Epidemiology Collaboration equation was used to calculate estimated glomerular filtration rate from serum creatinine for recipients and donors aged >18 years, and the modified Schwartz equation for those aged ≤18 years. Survival was estimated using the Kaplan–Meier method, and the log-rank test was employed for group comparisons.

**Results:**

A total of 229 kidney transplants in 221 patients were performed during the 20-year period, of which 135 (58.9%) were from LDs. Transplants carried out at LUH were 118 (51.5%), of which 116 were from LDs. During a median follow-up of 7.4 years (range 0.1–20), 27 (12.2%) patients died, 20 (74%) of whom had a functioning graft. One-year patient survival was 99.1% [95% confidence interval (CI), 97.9–100], 5-year survival was 95.7% (95% CI, 92.7–98.7), and 10-year survival was 87.7% (95% CI, 82.4–93.4). Death-censored graft survival was 98.3% (95% CI, 96.6–100), 96.8% (95% CI, 94.4–99.2), and 89.2% (95% CI, 84.1–94.7) at 1, 5, and 10 years, respectively.

**Conclusions:**

Patient and graft survival are comparable with those of large transplant centers, demonstrating the feasibility of running a quality kidney transplant program in a small nation in collaboration with a larger center abroad.

## Introduction

Kidney transplantation is the treatment of choice for the majority of patients with end-stage kidney disease (ESKD), resulting in better quality of life, greater survival, and lower cost when compared with maintenance dialysis (1, 2). Living donor (LDs) kidney transplantation results in superior outcomes compared with deceased donor (DD) transplantation (3, 4).

The first kidney transplantation in an Icelandic patient was a LD transplant performed in London in 1970. Over the ensuing three decades, all kidney transplants were performed at hospitals abroad, the majority at Copenhagen University Hospital. Deceased organ donation in Iceland was established in 1991 by passage of a legislation based on declaration of brain death and informed consent. Since then, organ procurement and transplantation services have been provided by a Nordic center (Copenhagen University Hospital in Denmark in 1997–2009 and the Sahlgrenska University Hospital in Gothenburg, Sweden, in 1992–1996 and since 2010) through a formal collaborative agreement. While the donation rate was low in the first 20 years, a multifaceted strategy, including a change in the law to an opt-out system in 2019, resulted in a marked increase in the donation rate, which over the past 5 years has averaged 22 per million population (5, 6). Expertise in transplant nephrology, tissue typing, and nephropathology gradually developed at Landspitali University Hospital (LUH) with the recruitment of professionals trained at transplant centers abroad. In 2003, a LD kidney transplant program was launched at LUH in close collaboration with an Icelandic transplant surgeon practicing in the United States who has performed the kidney transplants together with local surgeons. Beginning in 2019, a share of the DD kidney transplants has been carried out at LUH, depending on the availability of transplant surgeons. As less than 400,000 people reside in Iceland, kidney transplants are bound to be few each year. Hence, the decision to bring kidney transplantation to Iceland was controversial due to the small number of cases, while it was welcomed by patients and their families who had been forced to travel abroad to undergo the transplant surgery. In addition, the long distances may have resulted in prolonged cold ischemia time in case of DD kidneys, which is known to have a negative effect on both patient and graft survival (7). Despite advances in this area, a recent study showed that every additional hour(h) of cold storage time increases the risk of graft failure or death after transplantation (8).

Numerous studies over the past few decades have shown that the hospitals with the highest volume have better outcomes for major surgery (9). Consequently, several organizations and regulatory bodies have advocated for centralizing complex surgical procedures based on certain volume minimums (10, 11). However, published data on the impact of center volume on kidney transplantation outcomes have shown inconsistent results (12). Patient and graft survival after kidney transplantation has not been thoroughly investigated in Iceland, but doing so is important due to the small number of transplants performed locally.

In this study, we evaluated the outcomes of kidney transplantation in Icelandic patients over a 20-year period. Our aim was to compare both patient and graft survival to reported rates internationally to determine if our low-volume transplant program is justified.

## Material and methods

### Ethical approval

The study was approved by the Icelandic National Bioethics Committee (VSN 08-061) and the Icelandic Data Protection Authority.

### Study design and population

This retrospective observational cohort study included all Icelandic patients who underwent kidney transplantation between 1 January 2000 and 31 December 2019. In Iceland, the only center providing kidney care and transplant services is located at LUH, a regional hospital for the greater Reykjavík area and a tertiary referral center for the entire Icelandic nation. Between 2000 and 2009, organ procurement and transplantation of all DD kidneys were carried out by a team from Copenhagen University Hospital in Denmark, while the Sahlgrenska University Hospital in Gothenburg, Sweden, has provided these services since 2010. LD kidney transplants were performed at Copenhagen University Hospital until this service was brought to LUH in 2003. Icelandic patients have been placed on the kidney waiting list at the collaborating center and receive kidneys from either Icelandic or local donors according to allocation criteria. Icelandic recipients are immediately transported to the transplant center when a kidney becomes available, most commonly using a medical flight service. Patients have generally returned to Iceland within 1 or 2 weeks postoperatively. Pre-transplant evaluation and post-transplant care of DD kidney recipients has solely been provided at LUH. Commencement of DD kidney transplantation at LUH in 2019 was based on an agreement with the Sahlgrenska University Hospital Transplant Center stating that one or both kidneys procured from an Icelandic donor would be left behind for transplantation locally.

LUH became a member of Scandiatransplant when the first kidney transplant was performed at the hospital in 2003. Scandiatransplant, founded in 1969, is the Nordic organ allocation and exchange organization for Denmark, Estonia, Finland, Iceland, Norway, and Sweden, covering 11 kidney transplant centers and a population of 29.5 million (6). In addition to running a common transplant waiting list, Scandiatransplant operates a registry for donors, recipients, and the transplant procedures that includes follow-up data on transplanted patients and LDs.

The population of Iceland numbered 288,471 at the beginning of the study period on 1 January 2003 and had increased to 354,042 on 31 December 2019 (13). The population is primarily Caucasian with a genetic background which is a relatively homogeneous mixture of alleles from Scandinavia and the British Isles (14).

### Data collection

Information on patients was obtained from the Icelandic End-Stage Kidney Disease Registry (which includes all patients who have been treated with dialysis or kidney transplantation in Iceland since the first hemodialysis treatment was performed in 1968), medical records at LUH, and the Scandiatransplant database. Data collected included demographic information, body mass index (BMI), primary kidney disease, comorbid diseases (based on ICD-10 codes), dialysis before transplantation, other organ transplants, human leukocyte antigen (HLA) typing, panel reactive antibodies, cold and warm ischemia time, immunosuppressive medications, delayed graft function (DGF), viral studies, creatinine-based estimated glomerular filtration rate (eGFR), and causes of graft loss and death. Also obtained were donor-related variables, including BMI, creatinine-based eGFR, and ^51^Cr-EDTA plasma clearance.

### Assessment of kidney allograft function

The Chronic Kidney Disease Epidemiology Collaboration equation was used to calculate eGFR from serum creatinine for recipients and donors aged >18 years and the modified Schwartz equation for those aged ≤18 years. Serum creatinine values for kidney recipients and donors were obtained from an electronic laboratory database at LUH. Creatinine measurements were isotope dilution mass spectrometry (IDMS) standardized from 1 March 2008. Before this date, serum creatinine measurements were converted to IDMS standardized values. Based on methodology at the LUH Clinical Laboratory Services, measurements done before November of 2005 were converted using the following equation:

IDMSCr=(JaffeCr−11.834)/1.0355.

Between November 2005 and March 2008, the following equations were used:

$$\begin{array}{c} \mathrm{Jaffe}\;\mathrm{Cr}=\left(\mathrm{Roche}\;\mathrm{Enzymatic}\;\mathrm{Creatinine}\;\mathrm{Assay}+8.1\right)/1.02,\\ \mathrm{IDMS}\;\mathrm{Cr}\;=\;(\mathrm{Jaffe}\;\mathrm{Cr}-11.834)/1.0355. \end{array}$$

Average serum creatinine values were used for the determination of recipient eGFR for specific periods after kidney transplantation: 1 month, 2–3 months, 4–6 months, 7–12 months, and annually thereafter.

### Statistical analysis

Data are presented as number and proportion, mean ± standard deviation (SD) or median (range). The end of follow-up was 31 December 2020 or the time of graft loss or death if this occurred earlier. For patients who were lost to follow-up, the end of the follow-up period was the date of the last clinic visit. Patient and graft survival rates were estimated using the Kaplan–Meier method. Graft survival was defined as onset of maintenance dialysis or patient death, and the calculations were performed censored for death with a functioning graft. Comparison of groups was carried out using the Wilcoxon–Mann–Whitney test, Pearson chi-square test, and the log-rank test. We also performed a Cox regression analysis to estimate risk factors for graft loss, and a linear regression analysis to evaluate the association of various factors with recipient eGFR post-transplant. A *p*-value <0.05 was considered statistically significant. All analyses were carried out using RStudio version 1.1.423 (RStudio Team 2018; RStudio: Integrated Development for R. RStudio, Inc, URL: http://www.rstudio.com/).

## Results

A total of 229 kidney transplants were performed in 221 patients during the 20-year period, 135 (58.9%) of which were LD transplants. The number of transplants done at LUH was 118 (51.5%), of which 116 were from LDs. Other transplants were carried out abroad, mostly at Copenhagen University Hospital (between 2000 and 2009) and Sahlgrenska University Hospital (since 2010). The incidence of kidney transplantation increased markedly during the 20-year period. When divided into 5-year intervals, the incidence rose from 18 per million inhabitants in the first interval to 37, 41, and 46 per million inhabitants in the following intervals. The incidence was highest in 2018 (62 per million inhabitants) when 22 patients received a kidney transplant, 12 from DDs and 10 from LDs.

### Characteristics of kidney transplant recipients

Table 1 shows the characteristics of the 221 kidney graft recipients. In total, 29 (17.2%) patients underwent retransplantation, of whom 26 received their second and 3 patients their third kidney graft. As eight of these patients received their first and second kidney graft during the 20-year period, each transplant was treated as a separate case. Approximately 60% of the recipients were male patients. The median age was 47 years (range 3–76) and was significantly higher for recipients of DD grafts than LD grafts (*p* = 0.002). Of the recipients, 12 (5%) were aged ≤18 years. Of the LDs, 99 (73.7%) were biologically related to their recipients. Glomerular disease was the most common cause of kidney failure (36.7%), followed by cystic kidney disease (15.7%), while diabetic kidney disease was the cause in only 10.8% of cases. Comorbid conditions did not differ between donor types, except that recipients of DD grafts were more commonly affected by cardiovascular diseases other than coronary artery disease (*p* = 0.002). The baseline characteristics of the LD kidney graft recipients transplanted at LUH were similar to the whole group of LD kidney recipients.

**Table 1:** Baseline characteristics of kidney graft recipients, according to donor type.

Characteristic	Recipients of LD grafts (*n* = 135)	Recipients of DD grafts (*n* = 94)	*p*-value
Performed at LUH	116 (85.9)	2 (2.2)	
Sex, males	85 (63)	54 (57.4)	0.48
Age, years	45 (3–76)	52.5 (9–75)	**0**.**002**
Number of transplants per recipient			
1	121 (89.6)	79 (84)	0.15
2	14 (10.4)	12 (12.8)	0.57
3	0 (0)	3 (3.2)	
BMI, kg/m^2^	24.3 (14.6–40.8)	25.5 (14.1–37)	0.11
Primary kidney disease			
Glomerular disease	54 (40.0)	30 (31.9)	0.21
Cystic kidney disease	21 (15.5)	15 (16.0)	0.93
Tubulointerstitial disease	18 (13.3)	17 (18.1)	0.33
Hypertension	14 (10.4)	13 (13.8)	0.64
Diabetic kidney disease	12 (8.9)	12 (12.8)	0.35
Other and unknown	16 (11.9)	7 (7.4)	0.28
Comorbid conditions			
Hypertension	115 (85.2)	85 (90)	0.24
Coronary artery disease	31 (22.9)	22 (23.4)	0.94
Other cardiovascular disease	62 (45.9)	62 (65.9)	**0**.**002**
Diabetes	27 (20)	23 (24.4)	0.42
Time on waiting list, months	—	10.5 (0.1–96.3)	
Dialysis prior to transplantation	88 (65.2)	90 (95.7)	**<0**.**001**
Duration of dialysis, months	10 (0.5–203.7)	28.5 (0.5–132)	**<0**.**001**

BMI, body mass index; DD, deceased donor; LD, living donor; LUH, Landspitali University Hospital.

Continuous variables are shown as median (range) and categorical variables as number (%).

Bold values are statistically significant.

A total of 51 (22.3%) recipients underwent kidney transplantation as a pre-emptive procedure, 47 of whom received a LD graft and 4 received a DD graft. The median time on the kidney waiting list was only 10.5 months (range 0.1–96.3) for recipients of DD grafts. Among the remaining recipients, the median duration of dialysis was 17 months (range 0.3–203.7) and was significantly longer for recipients of DD grafts than LD grafts (*p* < 0.001). Only two patients died while on the waiting list and five patients were permanently delisted due to serious illness.

Transplant-related characteristics are shown in Table 2. There was a single case of ABO mismatch, where a recipient with blood group O received a kidney from a living A2 donor. Three or more HLA mismatches were present in 64.4% of the LD transplants and 77.7% of the DD transplants (*p* = 0.046), and the number of mismatches was significantly higher for DD grafts (*p* < 0.001). Eight recipients (8.6%) of DD grafts were considered highly immunized based on panel reactive antibodies (PRA) >80%. Seven patients received a simultaneous pancreas transplant, one of which never functioned. The median cold ischemia time of DD grafts was 14.4 h (range 1.3–44.9) and was longer than 24 h in 17 cases. Most recipients received induction therapy with basiliximab. The maintenance immunosuppressive regimen consisted of a combination of calcineurin inhibitor (tacrolimus >80%) and mycophenolate, and 71% of recipients were treated with steroids for at least 1 year after transplantation.

**Table 2:** Characteristics of kidney grafts at the time of transplantation, according to donor type.

Characteristic	Recipients of LD grafts (*n* = 135)	Recipients of DD grafts (*n* = 94)	*p*-value
ABO incompatibility	1 (1)	0 (0)	
HLA mismatch	3 (0–6)	4 (0–6)	**<0**.**001**
HLA mismatch ≥3 antigens	87 (64.4)	73 (77.7)	**0**.**046**
Anti-HLA antibodies			
PRA > 80%	—	8 (8.6)	
Other simultaneous solid organ transplantation			
Pancreas	0 (0)	7 (7.4)	**<0**.**001**
Heart	0 (0)	3 (3.2)	** **
Liver	1 (<1)	0 (0)	** **
Cold ischemia time, h	1.58 (0.8–3.5)	14.4 (1.3–44.9)	**<0**.**001**
Data missing	21	14	** **
Warm ischemia time, min	3 (1.25–7.2)	—	* *
Data missing	24	—	* *
CMV status (donor/recipient)			
D+/R+	103 (76.3)	50 (53.2)	* *
D+/R−	9 (6.7)	25 (26.6)	**<0.001**
D−/R−	3 (2.2)	6 (6.4)	*** ***
D−/R+	19 (14.1)	12 (12.8)	*** ***
Induction immunosuppressive therapy			
Basiliximab	99 (73.3)	58 (61.7)	**<0**.**001**
Alemtuzumab	13 (9.6)	1 (1.1)	** **
Thymoglobulin	4 (3)	8 (8.5)	** **
Data missing	19 (14.1)	27 (28.7)	** **
Maintenance immunosuppressive therapy			
Calcineurin inhibitors			
Tacrolimus	118 (87.4)	66 (70.2)	**0**.**0056**
Cyclosporine	11 (8.1)	20 (21.3)	** **
Anti-proliferative agents			
Mycophenolate	118 (87.4)	81 (86.2)	0.45
Azathioprine	3 (2.2)	4 (4.3)	
Both	12 (8.8)	6 (6.4)	** **
Steroids	80 (59.3)	83 (88.3)	**<0**.**001**

CMV, cytomegalovirus; D, donor; DD, deceased donor; HLA, human leukocyte antigens; LD, living donor; PRA, panel reactive antibodies; R, recipient.

Continuous variables are shown as median (range) and categorical variables as number and proportion (%).

Bold values are statistically significant.

### Kidney graft survival

The outcomes of kidney grafts are displayed in Table 3. In total, 45 (19.6%) grafts were lost during a median follow-up time of 7.4 years [range 0.13–20.7; 24 LD grafts after 8.3 (1.1–20.3) years and 21 DD grafts after 5.7 (0.13–20.7) years; *p* = 0.004]. A total of 20 (44%) recipients died with a functioning graft at a median of 8.1 years (range 0–20.3) after the transplantation. The median age at the time of death was 68 years (range 42–78). Malignancy was the most common cause of death (*n* = 6), followed by heart disease (*n* = 5). Four patients, all of whom received a DD kidney, died from an infection, including one patient who died from COVID-19. One patient died from kidney failure after declining dialysis treatment when graft failure occurred. Other causes of death were motor-neuron disease (*n* = 1) and suicide (*n* = 1), while the cause was unknown in two cases. One-year patient survival, censored for graft loss, was 99.1% [95% confidence interval (CI), 97.9–100], 5-year survival was 96.6% (95% CI, 94.0–99.4), and 10-year survival 89.6% (95% CI, 84.3–95.1)*.* Among the LD graft recipients transplanted at LUH, the 1-, 5-, and 10-year patient survival was 100%, 96.6% (95% CI, 93.0–100), and 90.2% (95% CI, 83.4–97.5), respectively.

**Table 3:** Kidney graft outcomes according to donor type.

Outcome	LD grafts (*n* = 135)	DD grafts (*n* = 94)	*p*-value
Follow-up time, years	8.3 (1.1–20.3)	5.7 (0.13–20.7)	**0**.**004**
Delayed graft function	0	21 (22.3)	**<0**.**001**
Recurrence of kidney disease	5 (3.7)	5 (5.3)	
eGFR, ml/min/1.73 m^2^			
Month 0–1 post-transplant (*n* = 201)	47 (9–133)	50 (15–191)	0.76
Month 7–12 post-transplant (*n* = 223)	62 (29–149)	57 (15–115)	**0**.**01**
Year 2–3 post-transplant (*n* = 187)	62 (11–103)	59 (14–133)	0.31
Year 4–5 post-transplant (*n* = 143)	59 (8–111)	57 (17–136)	0.84
Graft loss	24 (17.8)	21 (22.3)	0.39
Cause of graft loss			
Death with a functioning graft	10 (7.4)	10 (10.6)	
Chronic allograft nephropathy	9 (6.7)	6 (6.4)	
Recurrence of kidney disease	2 (1.5)	2 (2.1)	
Venous thrombosis	1 (<1)	1 (1.1)	
Primary non-function	1 (<1)	0 (0)	
Lack of treatment adherence	1 (<1)	1 (1.1)	
BK virus infection	0 (0)	1 (1.1)	

DD, deceased donor, eGFR, estimated glomerular filtration rate, LD, living donor.

Data are presented as number (%) or median (range).

Bold values are statistically significant.

Excluding those who died with a functioning graft, the most common cause of graft loss was chronic allograft nephropathy (36%). Three patients experienced early graft loss due to venous thrombosis (*n* = 2) or primary non-function (*n* = 1).

**Figure 1:**
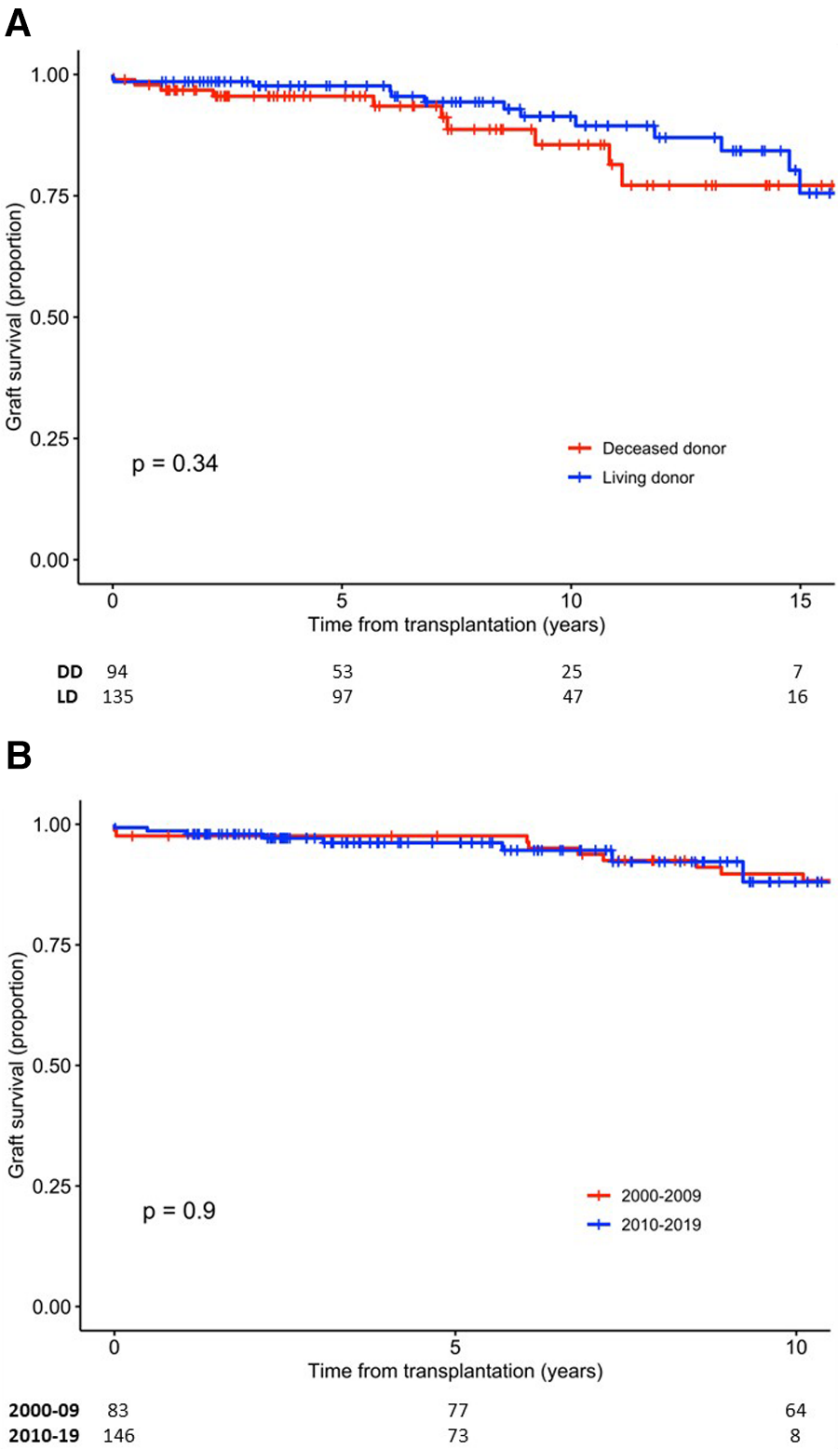
Survival of kidney grafts. (**A**) Graft survival according to donor type. (**B**) Graft survival according to time period of transplantation. DD, deceased donor; LD, living donor.

Death-censored graft survival was 98.3% (95% CI, 96.6–100), 96.8% (95% CI, 94.4–99.2), and 89.2% (95% CI, 84.1–94.7) at 1, 5, and 10 years, respectively. The survival of LD and DD grafts was comparable (*p* = 0.34) (Figure 1A), and no difference in graft survival was observed between the first and second half of the 20-year period (*p* = 0.9) (Figure 1B). Of the 116 LD kidney grafts transplanted at LUH, 17 were lost, which in 8 cases was due to death with a functioning graft. Graft survival for 1, 5, and 10 years, censored for death with a functioning graft, was 99.1% (95% CI, 97.5–100), 98.1% (95% CI, 95.6–100), and 93.6% (95% CI, 88.1–99.4), respectively.

### Risk factors for kidney graft loss

A total of 21 (22%) recipients of DD grafts experienced DGF, whereas this complication did not occur in any of the LD graft recipients. Recurrence of the original kidney disease occurred in 10 recipients.

The recipient eGFR at 7–12 months post-transplant, DGF and recurrence of the original kidney disease associated with the risk of graft loss (death censored) in a univariate Cox regression analysis (Table 4). For every 1 ml/min/1.73 m^2^ rise in the recipient mean eGFR at 7–12 months post-transplant, the risk of graft loss decreased by 3% (*p* = 0.03). Patients who experienced DGF or recurrence of kidney disease had more than a threefold increase in the risk of graft loss (*p* = 0.01 and *p* = 0.03, respectively). Due to a small sample size and a small number of lost grafts, it was not feasible to perform a multivariate analysis.

**Table 4:** Univariate Cox regression analysis of factors affecting the risk of death-censored kidney graft loss (*n* = 223, unless stated otherwise).

Variable	HR	95% CI	*p*-value
Recipient age, years	0.98	0.96–1.02	0.08
Age groups, years			
0–35	—	—	—
36–55	0.20	0.06–0.68	**0**.**01**
56–79	0.53	0.20–1.36	0.19
Recipient sex, female	0.94	0.42–2.10	0.89
Donor age, years	1.02	0.99–1.06	0.16
Donor sex, female	1.17	0.53–2.56	0.70
Donor type, deceased donor	1.47	0.67–3.27	0.34
Dialysis prior to transplantation	1.28	0.48–3.44	0.62
Retransplantation	1.8	0.67–4.81	0.99
Last donor eGFR before transplantation (*n* = 210)	0.99	0.97–1.01	0.42
Living donor ^51^Cr-EDTA plasma clearance before transplantation (*n* = 113)	1.00	0.98–1.033	0.55
HLA mismatch ≥3/6 antigens	0.70	0.32–1.57	0.16
Cold ischemia time, h (*n* = 80)	0.97	0.90–1.05	0.48
Delayed graft function	3.23	1.28–8.13	**0**.**01**
Most recent donor eGFR after transplantation (*n* = 132)	0.99	0.96–1.03	0.71
Recipient eGFR 7–12 months post-transplant (*n* = 223)	0.97	0.95–0.99	**0**.**03**
BK virus infection (PCR)	1.78	0.60–5.32	0.303
Recurrence of kidney disease	3.15	1.18–8.46	**0**.**03**
Time period			
2000–2004	—	—	—
2005–2009	0.39	0.14–1.06	0.07
2010–2014	0.73	0.24–2.24	0.59
2015–2019	0.22	0.02–2.06	0.18

eGFR, estimated glomerular filtration rate; HLA, human leukocyte antigens.

Hazard ratio for age groups and time period of kidney transplantation compares the groups to the first one. Calculations for cold ischemia only include deceased donors.

Bold values are statistically significant.

### Factors associating with recipient kidney graft function after transplantation

The relationship between selected donor- and recipient-related factors and median recipient eGFR at 7–12 months post-transplant was assessed using univariate linear regression (Table 5). Donor age, recipient age, preoperative donor eGFR, preoperative donor ^51^Cr-EDTA plasma clearance, recipient BMI, and DGF associated with the recipient eGFR.

**Table 5:** Univariate linear regression analysis of factors associated with recipient eGFR (ml/min/1.73 m^2^) 7–12 months after kidney transplantation (*n* = 223, unless stated otherwise).

Variable	Estimate (β)^a^	*P*-value
Recipient age, years	−0.54	**<0**.**001**
Recipient sex, female	5.17	0.09
Recipient pre-transplant BMI, kg/m^2^ (*n* = 210)	−1.54	**<0**.**001**
Donor age, years	−0.75	**<0**.**001**
Donor sex, female	−4.88	0.11
Donor preoperative BMI, kg/m^2^ (*n* = 207)	−0.16	0.61
Donor preoperative eGFR (*n* = 205)	0.40	**<0**.**001**
Donor preoperative ^51^Cr-EDTA plasma clearance (*n* = 111)	0.40	**<0**.**001**
Donor eGFR ≤3 days post-op (*n* = 107)	0.12	0.29
Mean donor eGFR 1 year after transplantation (*n* = 113)	0.18	0.23
Most recent donor eGFR (*n* = 130)	0.17	0.19
Dialysis prior to transplantation	5.51	0.13
Length of dialysis, months	−0.12	0.05
Retransplantation (*n* = 29)	4.24	0.35
HLA mismatch ≥3/6 antigens	−5.12	0.12
Cold ischemia time, h (*n* = 76)	0.52	0.11
Delayed graft function	−16.75	**<0**.**01**

BMI, body mass index; eGFR, estimated glomerular filtration rate; HLA, human leukocyte antigens.

^a^ The estimate indicates the change in median recipient eGFR 7–12 months after kidney transplantation. For continuous variables, an increase of 1 changes the eGFR by the observed estimate. For categorical variables, the estimate represents the difference in the mean eGFR between separate values of the variable. Assessment of recipient eGFR 7–12 months after transplantation was missing for six patients.

Bold values are statistically significant.

Again, it was not feasible to carry out multivariate regression that included all the predictor variables simultaneously due to a small sample size and missing data. Instead, we first entered four variables (donor and recipient age and sex) and subsequently added one variable at a time to the multivariate analysis (Supplementary Table S1). The predictor factors that were statistically significant in the univariate model also showed a statistically significant association with donor eGFR at 7–12 months post-transplant in the multivariate model.

## Discussion

The results of this study show that outcomes of LD kidney transplantation in Iceland are comparable to those at large transplant centers in other countries. Furthermore, the survival of kidney transplants from DDs performed abroad is satisfactory despite long journeys. Importantly, the cold ischemia time is relatively short and the incidence of DGF is acceptable. Our findings demonstrate the feasibility of running a quality kidney transplant program in a small country in collaboration with a larger center, provided that an experienced transplant surgeon who carries out a large number of transplants annually is a member of the team. The proportion of LD grafts is high in Iceland compared to other nations.

Approximately half of the kidney transplants were performed locally at LUH, after the establishment of the LD kidney transplant program 3 years into the study period. This initiative resulted in a rapid rise in the incidence of kidney transplantation, from 18 to 37 per million population between 2000–2004 and 2005–2009, and increased further to 46 per million in the last 5 years of the 20-year period. At the end of the study period, the incidence of kidney transplantation was higher than the average rate in Europe (15). As the incidence and prevalence of ESKD are low in Iceland compared to most other nations (16, 17), kidney transplantation as a treatment modality for ESKD is correspondingly high in Iceland. Indeed, 70% of patients receiving treatment for ESKD at the end of 2020 had a functioning kidney graft (16), while this rate is 40%–50% in many European countries (18).

Nearly 60% of kidney grafts in the current study were from LDs, which is high compared with most European countries where this rate is frequently <30% (15, 19, 20). The number of LD kidney transplants markedly increased after the kidney transplant program at LUH was established. The majority of LDs were female donors, as previously observed (21). LD kidney transplantation has several advantages for the recipient, including shorter waiting time, opportunity for a pre-emptive procedure, and avoidance of prolonged cold ischemia time. The time on dialysis before transplantation was much shorter for recipients of LD grafts, as other studies have shown (3, 22).

Patient and graft survival rates in Icelandic kidney transplant recipients are excellent when compared with other European countries and the USA (4, 19, 23). As most LD transplants were performed at our center, this finding demonstrates that a kidney transplant program can be successfully run despite a low volume of cases. Donors and recipients are carefully chosen according to standard criteria.

Studies examining the relationship between center volume and kidney transplant outcomes have shown conflicting findings (12, 24, 25). A meta-analysis by Tsampalieros et al. (12) did not demonstrate superior outcomes at large-volume centers, and the study by Sonnenberg et al., which includes the longest follow-up, suggests that transplantation center volume is not a substitute metric for outcome (25). Furthermore, the results of a systematic review suggest that high surgeon volume and specialization benefit patient outcomes, while the benefit of high hospital volume is less clear and varies between procedures (26). In general, regionalization is most effective for high-risk surgeries that have shown high variability in outcomes across hospitals (27). The drawbacks of regionalizing major surgery must be considered, including limitation of access to surgery, disruption of continuity of care and increased travel burden. The rationale of our approach included a high-volume transplant surgeon and the availability of transplant nephrology and expertise in necessary supporting services, such as immunology, pathology, infectious disease, as well as intensive care capacity and high-risk anesthesia. Nevertheless, we have avoided high-risk cases, such as HLA-incompatible transplantations, which are associated with a high risk of postoperative complications. Avoiding high-risk cases and closely monitoring outcomes may be particularly important in low-volume kidney transplantation programs.

The long-term outcome of kidney transplantation has been shown to depend on multiple factors related to the donor, the recipient, and the post-transplant period (28). Donor-related factors reflecting the quality of the kidney are especially important, including the type of donor, age, kidney function at the time of donation, and HLA compatibility. Among recipient-related factors are age, time spent on dialysis, recurrence of kidney disease, and post-transplant features, including acute rejection, DGF, and the immunosuppressive regimen. In the univariate analysis, recipient eGFR at 7–12 months post-transplant, DGF, and recurrence of the original kidney disease associated with the risk of graft loss. The eGFR as a marker of kidney graft function at 1 year has repeatedly been shown to be the best predictor of long-term graft outcome (28–31). This was indeed the case in our study, as for every 1 ml/min/1.73 m^2^ rise in the recipient mean eGFR at 7–12 months post-transplant, the risk of graft loss decreased by 3%. In our analysis, donor age, recipient age, preoperative donor eGFR, preoperative donor ^51^Cr-EDTA plasma clearance, recipient BMI, and DGF associated with recipient eGFR at 7–12 months post-transplant.

The median time on the waiting list among the recipients of DD grafts in our study was 10.5 months, which is shorter than has generally been reported in other countries (28–30). This finding, together with the high proportion of LD transplants, reflects good access to kidney transplantation for patients with ESKD in Iceland.

All but two DD transplants were carried out abroad, as these surgical procedures were not available in Iceland until 2019. The risk of prolonged cold ischemia time and DGF has been a concern due to the long-distance travel of the recipients. Interestingly, the median cold ischemia time in our study was only 14 h, which would be considered acceptable when compared to other studies (32–35). Moreover, the frequency of DGF in recipients of DD grafts was similar to that in previous reports (32, 36). While it is notable that no cases of DGF occurred in our group of LD transplant recipients, this may be due to chance in light of the small number of cases and low reported rates that are generally <5% (37, 38). As expected, DGF was associated with increased risk of graft loss.

It is also noteworthy that a cost-effectiveness analysis of our kidney transplant program carried out in 2009 showed that the total cost of LD transplantation locally proved to be significantly lower than for Icelandic patients transplanted in Copenhagen (39). The total cost per year of the care of each kidney transplant recipient was also much lower than the annual cost of dialysis treatment as shown by other studies (40).

The main strength of this study is the inclusion of all Icelandic patients who received a kidney transplant over a period of 20 years with follow-up data readily available for almost all patients. The main limitation of the study is the retrospective design. Some data were missing in part, for example, information on cold ischemia time. The small sample size, which is unavoidable in view of the size of the Icelandic population, and the excellent survival rate limited the analysis of risk factors.

In conclusion, our findings show that patient and graft survival rates in Icelandic kidney transplant recipients are comparable with those of large transplant centers in other countries, demonstrating the feasibility of running a quality transplant program in a small country in collaboration with a large center. The frequent use of LDs in Iceland when compared to other nations may partly explain our excellent results.

## Data Availability

The raw data supporting the conclusions of this article will be made available by the authors, without undue reservation.
