# Supplementary material for: Kidney transplantation in Icelandic patients, 2000–2019: are outcomes affected by low volume?
Source: Front Transplant. 2024 May 28;3:1398444. doi: 10.3389/frtra.2024.1398444 (PMC11235228; doi:10.3389/frtra.2024.1398444)
Supplement: Supplementary file 1 [file Table1.docx]

**Supplementary Table 1.** Multivariate linear regression analysis of factors affecting recipient eGFR (ml/min/1.73 m^2^) 7-12 months after kidney transplantation (n=223, unless stated otherwise).

| **Variable** | **Estimate (β)^a^** | **P-value** |
| --- | --- | --- |
| Recipient age, years | -0.34 | **<0.001** |
| Recipient sex, female | 2.86 | 0.28 |
| Recipient pre-transplant BMI, kg/m^2^ (n = 210) | -0.94 | **<0.01** |
| Donor age, years | -0.59 | **<0.001** |
| Donor sex, female | -3.4 | 0.19 |
| Donor preoperative BMI, kg/m^2^ (n = 207) | -0.03 | 0.89 |
| Donor preoperative eGFR (n = 205) | 0.21 | **<0.01** |
| Donor preoperative ^51^Cr-EDTA plasma clearance (n = 111) | 0.25 | **0.02** |
| Most recent donor eGFR after transplantation (n = 130) | 0.12 | 0.32 |
| Donor eGFR ≤3 days post-op (n = 107) | 0.07 | 0.69 |
| Mean donor eGFR 1 year post-op (n = 113) | 0.09 | 0.67 |
| Dialysis prior to transplantation | 4.15 | 0.18 |
| Length of dialysis, months | -0.07 | 0.24 |
| Retransplantation (n = 29) | 2.25 | 0.57 |
| HLA mismatch >3/6 antigens | -2.29 | 0.42 |
| Cold ischemia time, h (n = 76) | 0.05 | 0.86 |
| Delayed graft function | -15.78 | **<0.001** |

BMI, body mass index; eGFR, estimated glomerular filtration rate; HLA, human leukocyte antigens.

^a^The estimate indicates the change in median recipient eGFR 7-12 months after kidney transplantation. For continuous variables, an increase of 1 changes the eGFR by the observed estimate. For categorical variables, the estimate represents the difference in the mean eGFR between separate values of the variable. The top four variables in the table were first entered into the model and the other variables subsequently added, one at a time. Assessment of recipient eGFR 7-12 months after transplantation was missing for 6 patients.

Bold values are statistically significant.
